# Optimal waist circumference cut-off points for predicting metabolic syndrome among low-income black South African adults

**DOI:** 10.1186/s13104-018-3136-9

**Published:** 2018-01-12

**Authors:** Eyitayo Omolara Owolabi, Daniel Ter Goon, Oladele Vincent Adeniyi, Anthony Idowu Ajayi

**Affiliations:** 1 Department of Nursing Science, Faculty of Health Sciences, University of Fort Hare, East London, South Africa; 2 Department of Family Medicine, Faculty of Health Sciences, Walter Sisulu University, Cecilia Makiwane Hospital, East London Hospital Complex, East London, South Africa; 3 Department of Sociology, Faculty of Social Science and Humanities, University of Fort Hare, East London, South Africa

**Keywords:** Metabolic syndrome, Abdominal obesity, ROC curves, Youden index, Africans, South Africans

## Abstract

**Objective:**

Waist circumference has been identified as one of the strongest predictive tool for metabolic syndrome. This study determines the optimal cut-off point of waist circumference for metabolic syndrome among low-income earning South African black population, in Eastern Cape, South Africa. The optimal waist circumference cut-off point was determined through receiver operating characteristics analysis using the maximum Youden index.

**Results:**

Among men, waist circumference at a cut-off value of 95.25 cm yielded the highest Youden index of 0.773 (sensitivity = 98%, specificity = 79%, area under curve 0.893). For women, waist circumference of 89.45 cm yielded the highest Youden index of 0.339 (sensitivity = 88%, specificity = 46%, area under curve 0.713). The prevalence of metabolic syndrome among men, women and both sexes using the new cut-off points were: 17.8, 20.8 and 17.7%, respectively, compared to; 15.6, 24.8 and 21.8%, using the traditional cut-off values of 94 and 80 cm for men and women, respectively. The traditional waist circumference value slightly under-estimated the prevalence of metabolic syndrome among men and over-estimated among women and the overall population. A specific waist circumference cut-off point for South African blacks is needed for correct identification of the metabolic state of the populace in order to develop appropriate interventions.

**Electronic supplementary material:**

The online version of this article (10.1186/s13104-018-3136-9) contains supplementary material, which is available to authorized users.

## Introduction

Metabolic syndrome (METs) is characterized by clustering of metabolic abnormalities such as central obesity, hypertension, dyslipidemia, and glucose intolerance [1]. It is an essential public health challenge as a result of its association with cardiovascular disease, a leading cause of morbidity and mortality [2]. The waist circumference (WC) has been identified as one of the strongest predictive tool for METs. This anthropometric indicator is easy, inexpensive and non-invasive and can be applied in clinical practice. However, due to the absence of country specific WC cut-offs, the validated WC cut-points (≥ 94 cm in men and ≥ 80 cm in women) for European populations are presently being utilised by researchers and clinicians in screening for abdominal obesity in African and African-descent individuals [3]. This is not optimal, as dichotomy exist in the pattern of fat distribution between Caucasians and black Africans [4, 5]. Also, several studies have suggested that the METs as currently defined may not be appropriate to predict cardiovascular disease and type 2 diabetes risk in Africans [6–8]; as Black Africans exhibit more propensity to insulin resistance, and a higher prevalence of hypertension and low high density lipoprotein cholesterol (HDL-C) levels, in contrast to lower rates of hypertriglyceridemia compared to the Caucasians [9, 10].

Controversies exist concerning the correct anthropometric values relative to ethnicity, genetic background, sex, and sociocultural context [11]. Studies have shown that WC is among the most powerful tools for predicting METs and that the optimal cut-off values for various indices, including WC, may differ by sex and race [12–16]. Studies investigating the WC cut-points of METs for Africans are rare, and have reported inconsistent results [17–19]. Additionally, such studies in the South African context are conducted in high-income and urban settings [17, 19–23]; while only few studies are reported in low-income, rural settings [18, 24]. We sought to determine the optimal cut-point of WC for METs among low-income earning, rural South African black population, using the cardiometabolic screening data of out-patients attending health facilities in Buffalo City Metropolitan Municipality, Eastern Cape, South Africa.

## Main text

### Methods

Detailed accounts of the sampling procedures for cardiometabolic screening data have been published elsewhere [25–27]. Briefly, this cardiometabolic screening survey was conducted at the three largest out-patient clinics in the largest settlement in Buffalo City Metropolitan Municipality, South Africa. A sample size of 1107 participants was estimated across the three study sites (369 per site), based on the estimated non-communicable disease prevalence rate of 40% in South Africa, with a sampling error of 5 and a 95% confidence level. Due to incomplete data, 109 participants were excluded, thus, only 998 adults (321 males, 627 females) were included in the analysis. Eligibility criteria included age ≥ 18 years, attendance at the out-patient clinics, and 8 h of fasting prior to recruitment into the study. Patients who were psychotic, debilitated, pregnant or handicapped in any form to the point that obtaining anthropometric measurement would be difficult were excluded from the study. All ambulatory individuals who fulfilled the inclusion criteria and attended the study settings during the period of study were recruited into the study. This study was conducted in April and May, 2016. A convenience sampling method was utilised.

Participants were interviewed using the previously validated WHO STEPwise questionnaire which comprises three major items; demographic data, behavioural data and measurements. The questionnaire was written in english. Interview was conducted by trained research assistants and both anthropometric and blood pressure measurements were done by trained professional nurses. Waist circumference, blood pressure and fasting blood glucose measurements followed standardised protocols. Metabolic syndrome was defined using the International Diabetes Federation (IDF) criteria as the presence of any three of the following five criteria; high blood pressure, diabetes, prediabetes, high cholesterol and abdominal obesity [28].

Data were analysed using Statistical Package for Social Sciences (SPSS) software, version 23.0 (SPSS Inc. Chicago, IL). The optimal WC cut-point was determined through Receiver Operating Characteristics (ROC) curve analysis using the Youden index [maximum (sensitivity + specificity − 1)] [29]. Waist circumference was excluded from the classification of METs, because it was an outcome variable for developing cut-points. Previous studies have used two or more components other than WC to classify METs in South Africa [18, 22]. Analysis was done at a confidence interval of 95%. A p  < 0.05 was considered statistically significant.

### Results

The mean age of participants was 42.6 (SD ± 16.5) years. The majority of the participants were black (98.1%), female (67.8%), single (63.9%) and had at least secondary (grade 8) level of education (69.7%). About half of the participants had no income (44.6%) and were unemployed (47.7%), while only a few (7.5%) participants earned above 400USD monthly (Additional file 1: Table S1).

**Fig. 1:**
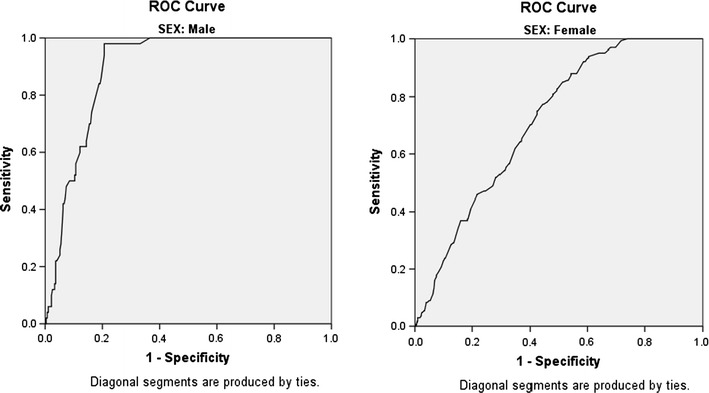
ROC curve for waist circumference as a predictor of metabolic syndrome among men and women

**Fig. 2:**
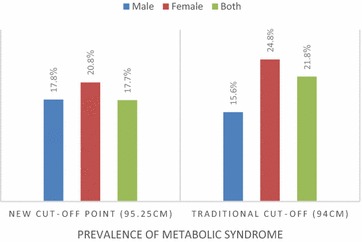
Prevalence of metabolic syndrome using the new and the traditional cut-off points

Among men, WC at a cut-off value of 95.25 cm yielded the highest Youden index (0.773) with a corresponding sensitivity of 98% and specificity of 79% [area under the ROC curve (AOC) 0.893, p  < 0.001, 95% confidence interval (CI) 0.858–0.928]. At the traditional cut-off value of 94 cm, the Youden index slightly dropped to 0.74, with sensitivity and specificity remaining the same. For women, the WC at a cut-off value of 89.45 cm yielded the highest Youden index (0.339) with a sensitivity of 88% and specificity of 46% (AOC 0.713, p  < 0.001, 95% CI 0.673–0.753). At the traditional cut-off value of 80 cm, the Youden index dropped to 0.249 with a corresponding increase in sensitivity to 100% and a significant reduction in specificity to 25% (Fig. 1). The prevalence of METs among men, women and both sexes using the new cut-points (WC ≥ 95.25 cm for men and ≥ 89.45 cm for women) were: 17.8, 20.8 and 17.7%, respectively, compared to; 15.6, 24.8 and 21.8%, using the traditional cut-off values of 94 and 80 cm for men and women, respectively (Fig. 2).

### Discussion

To the best knowledge of the authors, this is the first study to determine the optimal WC cut-points for predicting METs among low-income black South African adults in the Eastern Cape region. An earlier study reported a WC of 91.5 cm in diagnosing METs among urban South African women in Soweto [17]; while another study reported a WC of 92 cm as optimal for women [18]; and yet, a WC of 98 cm for African women has been reported [19]. In this present study, the prevalence of METs among men, women and both sexes using the new cut-off points (WC ≥ 95.25 cm for men and ≥ 89.45 cm for women) were: 17.8, 20.8 and 17.7%, respectively, compared to; 15.6, 24.8 and 21.8%, using the traditional cut-off values of 94 cm and 80 cm for men and women, respectively. This confirms the inconsistencies in WC cut-points in different races and sexes as reported in the literature [12–16]. The optimal cut-point of WC for the diagnosis of metabolic syndrome is distinct in different African countries (Table 1).

**Table 1:** Comparison of optimal cut-off point of waist circumference (WC) for the diagnosis of metabolic syndrome in African countries with the present study

Country [references]	n	Cut-off point for men (cm)	Cut-off point for women (cm
Uganda [24]	6136	≥78 to ≥ 80	≥ 82 to ≥ 85
South Africa [19]	123	90	98
Nigeria [36]		76	72
Cameroon [36]		81	82
Benin [23]	452	80	94
Angola [31]	615	87.5	80.5
South Africa [18]	947	86	92
Benin [37]	541	80	90
South Africa [17]	1251	91.5	
South Africa [21]	152	92	94
South Africa [38]	1099	84	94
South Africa [22]	920	87.5	94.6
South Africa [20]	203	≥ 88 or ≥ 90	–
South Africa (present study)	998	≥ 95.25	≥ 89.45

Notwithstanding the differences in defining METs, the WC cut-off points are persistently high in African women. This could be explained by the high prevalence of obesity among women compared to men in the African societies, shaped by socio-economic and environmental variables. Given that women have higher proportion of total subcutaneous fat distribution compared to men [30], the potential risk of misclassification of women as having excessive visceral adiposity by using values of WC to predict other components of the METs [31], cannot be ignored. Conversely, Lemieux [32] and Alberti [33] reports a low WC cut-off in men compared to women, which was attributed to the notion that on average, men have twice as much visceral abdominal fat than premenopausal women [32].

Notably, consistent with our study, the waist WC cut-point currently being utilised for the diagnosis of METs in sub-Saharan African females (80 cm) [34, 35] is too low and will therefore over-estimate the prevalence of METs. As such, there is need to validate the WC ≥ 95.25 cm for men and ≥ 89.45 cm for women observed in this present study for other sub-Saharan African populations. Country, ethnic-and gender-specific WC cut-off points are needed, because adopting other WC criteria to diagnose African black populations may either under-or overestimate the presence of METs.

As pointed by Murphy et al. [24], optimal ethnic-specific WC cut-off points are seemingly useful as a screening tool that provides benefits in the detection of obesity and assessing the risks of other related diseases such as diabetes and cardiovascular disease. Viewed in this perspective, the findings from this prospective study provide up-to-date, evidence-based data that can be utilised for public health interventions in low-income populations, at least among underserved black Africans, in this setting.

### Conclusion

The traditional waist circumference value used for the diagnosis of metabolic syndrome may not be suitable for this study participants as it might have slightly under-estimated the prevalence of METs among men and over-estimated the prevalence of METs among the women and the overall population. There is a need to determine a specific WC cut-off point for South African blacks as this will assist in correctly identifying the metabolic state of the populace and develop appropriate interventions.

## Limitations

Using measured anthropometric variables, as opposed to self-reported values, in a relatively large sample with very reliable data are the main strengths of this study. The limitations of the study should be noted.

- Due to the cross-sectional nature of the study, we therefore cannot infer causality. The WC cut-off points proposed by our study need to be confirmed in a longitudinal study.
- In addition, we did not measure the lipid levels in the study sample which might have underestimated the burden of METs in the setting.

## Additional file

**Additional file 1: Table S1.** Demographic characteristics of the study participants.

## Abbreviations

WC: waist circumference
METs: metabolic syndrome
HDL: high-density lipoprotein
ROC: receiver operating characteristics
AOC: area under receiver operating characteristics curve
IDF: International Diabetes Federation
