# Supplementary material for: Optimal waist circumference cut-off points for predicting metabolic syndrome among low-income black South African adults
Source: BMC Res Notes. 2018 Jan 12;11:22. doi: 10.1186/s13104-018-3136-9 (PMC5766973; doi:10.1186/s13104-018-3136-9)
Supplement: Supplementary file 1 — Additional file 1: Table S1. Demographic characteristics of the study participants. [file 13104_2018_3136_MOESM1_ESM.docx]

**Table S1: Demographic characteristics of the participants**

| Variables | Male (n=321)  n(%) | Female (n=677)  n(%) | Total (n=998)  n(%) | p-value |
| --- | --- | --- | --- | --- |
| Age group (years) |  |  |  |  |
| 18-25 | 40(12.5) | 143(21.1) | 183(18.3) |  |
| 26-35 | 74(23.1) | 149(22.0) | 223(22.3) |  |
| 36-45 | 67(20.9) | 116(17.1) | 183(18.3) | 0.009 |
| 46-55 | 57(17.8) | 110(16.2) | 167(16.7) |  |
| 56-65 | 41(12.8) | 99(14.6) | 140(14.0) |  |
| ≥66 | 42(14.1) | 60(8.9) | 102(10.2) |  |
| Level of education |  |  |  |  |
| No formal schooling | 62(19.3) | 84(12.4) | 146(14.6) |  |
| Grade 1-7 | 57(17.8) | 99(14.6) | 156(15.6) | 0.008 |
| Grade 8-12 | 171(53.3) | 409(60.4) | 580(58.1) |  |
| Tertiary | 31(9.7) | 85(12.6) | 116(11.6) |  |
| Monthly income (Rands) |  |  |  |  |
| No income | 134(41.7) | 300(44.3) | 445(44.6) |  |
| R150-2000 | 89(27.7) | 248(36.6) | 326(32.7) | 0.000 |
| R2001-5000 | 74(23.1) | 100(14.8) | 174(17.4) |  |
| R5001and above | 24(7.5) | 29(4.3 | 53(5.3) |  |
| Marital status |  |  |  |  |
| Single | 193(60.3) | 444(65.6) | 637(63.9) |  |
| Married | 115(35.9) | 185(27.3) | 300(30.1) |  |
| Separated | 1(0.3) | 5(0.7) | 6(0.6) | 0.002 |
| Divorced | 9(2.8) | 13(1.9) | 22(2.2) |  |
| Widowed | 2(0.6) | 30(4.4) | 32(3.2) |  |
| Racial group |  |  |  |  |
| Black | 313(97.5) | 666(98.4) | 979(98.1) |  |
| Coloured | 8(2.8) | 9(1.3) | 17(1.7) | 0.026 |
| White | 0(0.0) | 2(0.3) | 2(0.2) |  |
| Type of employment |  |  |  |  |
| Government employee | 30(9.3) | 33(4.9) | 63(6.3) |  |
| Non-government employment | 98(30.5) | 133(19.7) | 231(23.2) |  |
| Self-employment | 30(9.3) | 32(4.7) | 62(6.2) |  |
| Students | 19(5.9) | 80(11.8) | 99(9.9) | 0.000 |
| Unemployed | 115(35.8) | 361(53.4) | 476(47.7) |  |
| Retired | 29(9.0) | 37(5.5) | 66(6.6) |  |
